# Relationship between Air Pollution and Regional Longevity in Guangxi, China

**DOI:** 10.3390/ijerph16193733

**Published:** 2019-10-03

**Authors:** Qucheng Deng, Yongping Wei, Lijuan Chen, Wei Liang, Jijun Du, Yuling Tan, Yinjun Zhao

**Affiliations:** 1 School of Earth and Environmental Sciences, The University of Queensland, Brisbane 4072, Australia; uqqdeng@uq.edu.au (Q.D.); yongping.wei@uq.edu.au (Y.W.); 2 Key Laboratory of Ecohydrology of Inland River Basin, Northwest Institute of Eco-Environment and Resources, Chinese Academy of Sciences, Lanzhou 730000, China; 3 Guangxi Environmental Information Center, Nanning 530028, China; liangwei_gxepb@163.com; 4 Chinese Research Academy of Environmental Sciences, Beijing 100101, China; dujijun2007@163.com (J.D.); tanyuling11@163.com (Y.T.); 5 Key Laboratory of Environment Change and Resources Use in Beibu Gulf, Ministry of Education, Nanning Normal University, Nanning 530001, China; crpp0104@163.com

**Keywords:** longevity, air pollutants, geographically weighted regression, Guangxi, Hechi

## Abstract

Air pollution has become a global environmental challenge and poses major threats to human health, particularly for the aging population. However, few studies have investigated the effects of air pollutants on human longevity, especially based on the total regional quantities and sources. Based on investigation of the spatiotemporal variations of three air pollutants (PM_10_, SO_2_, and NOx) and three longevity indicators (centenarian ratio, centenarity index, and aging tendency), this study aims to identify the relationship between air pollution and regional longevity in Guangxi Province. Air pollutant and population data from 109 counties and areas of Guangxi were collected from environmental research reports and statistical yearbooks. Cluster and outlier analysis was used to detect the regions with high and low clusters of the longevity indicators and air pollutants. Geographically weighted regression analyses were performed to determine the relationship between longevity and air pollutants. A negative relationship between the air pollutants PM_10_, SO_2_, and NOx on the aged population was observed. From a provincial level, industrial sources from the urban areas of cities located in the central province, including Liuzhou, Nanning, Laibing, Guigang and Yulin, were important contributors to the air pollutants PM_10_, SO_2_, and NOx, and thus could contribute to negative impacts on regional longevity. The key findings from this study will provide a case for management of air pollutants based on public health policies in China as well as other developing communities.

## 1. Introduction

Aging has imposed increasing challenges on global communities due to its considerable socioeconomic impacts [1]. The aging population has weakened immune systems and hypofunction and this group could be impacted by socioeconomic [2,3], medical [4], genetic [5,6], and environmental factors [7,8]. The impacts of socioeconomic and environmental factors on the health of the aging population have been receiving increasing attention.

Generally, aging can be described by different longevity indicators, with the centenarian ratio, centenarity index, and aging tendency the most widely used indicators [7,8,9,10]. The centenarian ratio and centenarity index are very important indicators for measuring the extreme longevity in a given area. The centenarity index could reflect the extreme longevity and significantly exclude the effects of the local birth rate and the proportion of immigrants [9]. For example, Wang et al. used the centenarian ratio as an indicator to identify areas with higher longevity population in China from 2000 to 2010 and examined the related influential factors [2]. Liu et al. used the centenarity index to represent the regional longevity level in Xinjiang and examine how it is associated with major parameters in drinking water [11]. The aging tendency is generally representative of the local aging condition. 

Air pollutants could have negative impacts on human health. SO_2_, NOx, PM_10_, PM_2.5_, O_3_, and CO have been intensively researched via air quality assessments. Among these indicators, SO_2_, NOx, and PM_10_ are considered the major monitored air pollutants. Inhalation of air polluted with these substances can impact human health [12,13], such as cardiovascular [14] and respiratory systems [15] as well as triggering other diseases [16,17,18,19,20,21] which could indirectly reduce human life expectancy. Wong et al. evaluated the effects of air pollution on public health among some major Asian cities and found that NOx, SO_2_, PM_10_, and ozone affected human health by increasing the vulnerability to chronic diseases, such as cardiovascular and respiratory mortality [22]. Raaschou-Nielesen et al. studied the relationship between lung cancer incidence and air pollution in 19 European regions, and found links between pollution levels (PM_10_ and PM_2.5_) and the hazard ratio for lung cancer [23]. Bowe et al. examined the relationship between PM_2.5_ and the national burden of diabetes mellitus from a global perspective and found that increased PM_2.5_ levels were associated with an increased risk for diabetes [24]. 

Although many studies have focused on the association between specific air pollutants and impacts on human health, limited few examined the relationship between air pollutants and the longevity indices. From these examples, Apte et al. examined the relationship between an ambient PM_2.5_ concentration and diseases associated with human longevity in 185 countries and found that PM_2.5_ could reduce human life expectancy, especially in developing countries [25]. Wang et al. examined the relationship between the longevity ratio and air pollutants SO_2_ and PM_10_ in 85 major cities of China and found that an increase in both air pollutants could have contributed to a decrease in longevity, with PM_10_ having a more significant impact [26]. Similarly, Song et al. examined the association between longevity indicators regarding centenarian ratio, longevity index, and socio-economic and environmental factors among provinces in China and concluded that SO_2_ and PM_10_ increase could decrease the regional longevity [27]. However, there are fewer studies established links between the total amount of these air pollutants, their source composition and regional longevity, and thus failed to provide direct guidance for air pollution control from its origin to improve public health management for the aging population. 

This study aims to investigate the links among air pollutants, their source composition, and regional longevity in Guangxi Province, with a special focus on Hechi, a longevity city which has been recognized due to its leading centenarian ratio and the number of centenarians. Based on the data of 109 counties and areas, the spatial distribution of PM_10_, SO_2_, and NOx were analyzed, and the source composition of each air pollutant was also examined. Finally, the statistical relationships between air pollutants and longevity indicators was investigated. 

## 2. Materials and Methods

### 2.1. Study area Description

**Figure 1:**
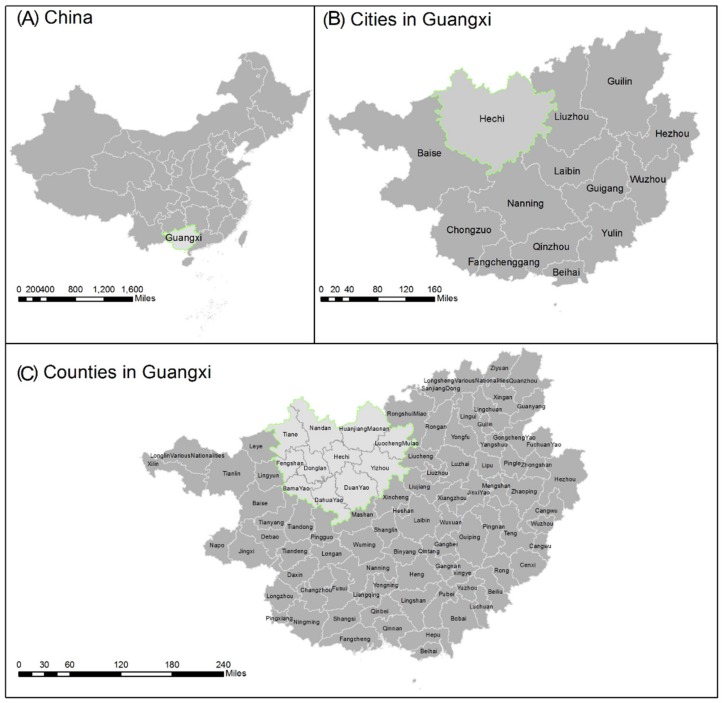
Case study of Guangxi (**B**), (**C**) in China (**A**).

Guangxi Province is located at a low latitude (20°54’–26°24’ N, 104°28’–112°04’ E) in Southern China, covering an area of approximately 237,600 km^2^ (Figure 1). Because of its considerable elderly population, diverse subtropical climate and topography, and different levels of industrialization and economic development, Guangxi Province could be an ideal site for investigating the relationship between air pollution and regional longevity. 

Most of the plain areas within Guangxi Province are situated in the southern and middle areas, and the mountainous areas are mainly located in the northwestern area. Guangxi has 14 cities, which contain 109 counties and areas in total (Figure 1) and have a population of approximately 51 million [28]. The 14cities in Guangxi are: Nanning, Liuzhou, Guilin, Laibing, Yulin, Guigang, Fangchengang, Qinzhou, Hechi, Baise, Chongzuo, Hezhou, Wuzhou, and Beihai. In 2010, the gross domestic product (GDP) in Guangxi was approximately 9570 billion yuan [28]. The population distribution, economic development, and industrialization in Guangxi have been uneven. Nanning is the capital city and the economic center of Guangxi (Figure 1), and it has a relatively higher economic and industrial scale. Liuzhou and Laibin are important industrial cities in Guangxi and have higher industrialization. Guigang, Yulin, Beihai, Qinzhou, and Fangchenggang, which are located in the southern areas of Guangxi, have a dense population, higher industrialization, and a relatively developed economy. The longevity city Hechi is located in the northwestern area of Guangxi, and for decades, it has had a relatively low economy and industrialization percentage compared with the other cities in Guangxi [10,29].

Guangxi was recognized as a province with the highest centenarian ratio in mainland China based on the Sixth National Population Census. Guangxi contains a substantial population of individuals over 65 years of approximately 3.9 million [30]. Hechi was identified as a longevity city due to its leading centenarian ratio (17.9/100,000) in 2016 [29], which is higher than the longevity areas defined by the United Nations (7/100,000), and it contains 11 counties (Bama, Dahua, Donglan, Duan, Fengshan, Huanjiang, Jinchengjiang, Luocheng, Nandan, Tian’e, and Yizhou) with Bama, Dahua, Yizhou, Donglan, Fengshan, and Tian’e all recognized as “longevity counties”, due to the substantial ratio of centenarians in those areas [29].

### 2.2. Data Collection of the Air Pollution Sources and Population Data Collection

The primary data of air pollutants and air pollution sources were obtained from the First National General Survey of Pollution Sources [31,32,33,34]. The first National General Survey of Pollution Sources was the first national comprehensive investigation on the air pollutants in China, and the data were in the format of on-site investigations, census pilots, quality control, and data reviews conducted in an integrated way to ensure the reliability and accuracy of the results. This survey was jointly released by the Ministry of Environmental Protection of China, the National Bureau of Statistics, and the Ministry of Agriculture and Rural Affairs of China in 2011. This is the most updated survey information on pollution sources in China.

Based on the National General Survey of Pollution Sources, the three major air pollutants (PM_10_, SO_2_, and NOx) and their sources, including industrial, civil points, and mobile, in Guangxi were published at a scale of 10 km × 10 km in 2011 [35]. This survey is the data source for the three air pollutants PM_10_, SO_2,_ and NOx and their source composition in the 109 counties and areas in Guangxi examined in this study. 

The population data of this study were obtained from the Sixth National Population Census in 2010. With the support of the United Nations Population Fund in 1982, the Chinese government introduced rules in the census, such as age verification, to control the quality of the survey data. Many academic and organizational experts have recognized the accuracy and reliability of the Chinese censuses [36]. This is the most updated survey information on population in China.

Three main indicators were used in this study: centenarian ratio, centenarity index and aging tendency. The centenarian ratio represents the percentage of centenarians per 100,000 people and is an important and frequently used indicator for measuring the longevity phenomenon. The centenarity index represents the percentage of inhabitants older than 100 years old to the inhabitants older than 90 years old to reveal the percentage of the relatively elderly population [9]. This indicator could reflect the percentage of the extreme elderly population while excluding the factors birth rate and immigrant status [9]. The aging tendency refers to the percentage of people over 65 years old to the total population, and it has been widely used to measure the tendency of the local aging condition. Thus, using the three longevity indicators could comprehensively reflect the extreme longevity and general aging conditions. 

### 2.3. Data Analysis 

The descriptive analysis in this study was conducted using SPSS 22.0 (IBM, New York, NY, USA), and the geographic distribution maps of the air pollutants, air pollution sources (PM_10_, SO_2_, and NOx), and longevity indicators (centenarian ratio, centenarity index, and aging tendency) were generated using ArcGIS 10.5.1 (ESRI, Redlands, CA, USA). Then, the cluster and outlier analysis was employed by ArcGIS 10.5.1 to determine the high and low cluster areas of both the longevity indicators and the air pollutants [37,38]. Before the statistical analysis was conducted, we employed the Kolmogorov–Smirnov test to determine whether the data were normally distributed, and if not, a logarithmic transformation was used to normalize the data. Statistical relationship between three air pollutants and three longevity indicators individually were performed using geographically weighted regression in ArcGIS 10.5.1. [38,39]. 

## 3. Results

### 3.1. Spatial Distribution and Clustering of the Three Air Pollutants in Guangxi

Significant differences were found in the distribution of SO_2_, NOx, and PM_10_ across the 109 counties and areas in Guangxi Province (Table 1). The average concentration of SO_2_ was 2329 tons per annum (t/a), and the standard deviation was 3683 t/a, thus showing a significant difference among the counties. Similarly, the average emissions and standard deviation were 1839 t/a and 3060 t/a for NOx, respectively, and 2061 t/a and 2013 t/a for PM_10_, respectively.

**Figure 2:**
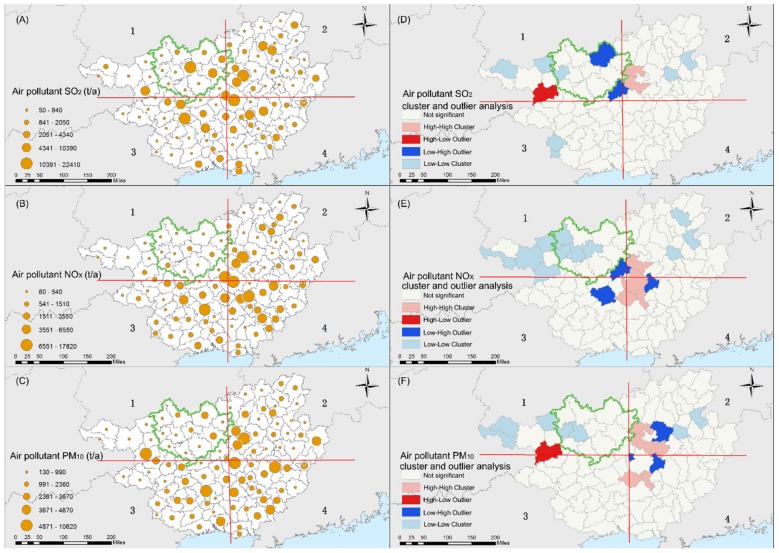
Spatial distribution of the three air pollutants (**A**–**C**) and the results (**D**–**F**) of the cluster and outlier analysis.

The highest values of SO_2_, PM_10_, and NOx were detected in central Guangxi (Figure 2), mainly in large cities with better industrialization and economy, such as Liuzhou, Laibing, and Nanning. In the urban area of Liuzhou, the levels of SO_2_ ranged from 10,391 to 22,410 t/a, which represented the highest SO_2_ pollution in Guangxi. Similarly, the highest PM_10_ and NOx pollution also appeared in the urban areas of Liuzhou. Baise City and Hechi City had moderate SO_2_ air pollutants (Figure 2A). Inside Hechi City, the SO_2_ emissions were mainly distributed in the northeastern sections (Hechi urban areas, Yizhou and Luocheng). In Dahua, Donglan, and Bama counties, which are located in the southwestern areas of Hechi City, the lowest SO_2_ pollution source levels were observed (Figure 2A), with values of 80 t/a, 130 t/a, and 140 t/a, respectively. The counties with the lowest levels of NOx were Donglan, Fengshan, and Dahua (Figure 2B), with values of 130 t/a, 140 t/a, and 170 t/a, respectively. The levels of PM_10_ in Donglan, Fengshan, and Bama of Hechi, Tianlin and Longlin of Baise, and Yangshuo of Guilin, were the lowest (Figure 2C) at 130 t/a, 230 t/a, 230 t/a, 130 t/a, 160 t/a, and 170 t/a, respectively. 

The cluster and outlier analysis showed that the areas with high clusters of SO_2_ were located in central urban areas of Liuzhou, the northwestern area of Liuzhou including the counties of Liucheng and Liujiang, whereas the low clusters were located in Fengshan in Hechi City and Longlin and Leye in Baise City (Figure 2D). Meanwhile, the areas with high clusters of PM_10_ and NOx emissions were mainly distributed in the central area (Figure 2E–F), including Nanning, Liuzhou, Laibing, and Guigang cities, and the areas with low clusters were mainly distributed in northwest Guangxi, especially in the western areas of Hechi City, such as Bama, Donglan, Fengshan, and Tian’e with lower clusters of NOx, whereas in Donglan and Fengshan, with lower clusters of PM_10_, small clusters of SO_2_, PM_10_, and NOx emissions were observed, demonstrating relatively good air quality conditions. 

### 3.2. Spatial Distribution and Clustering of the Source Compositions of Air Pollutants in Guangxi

**Figure 3:**
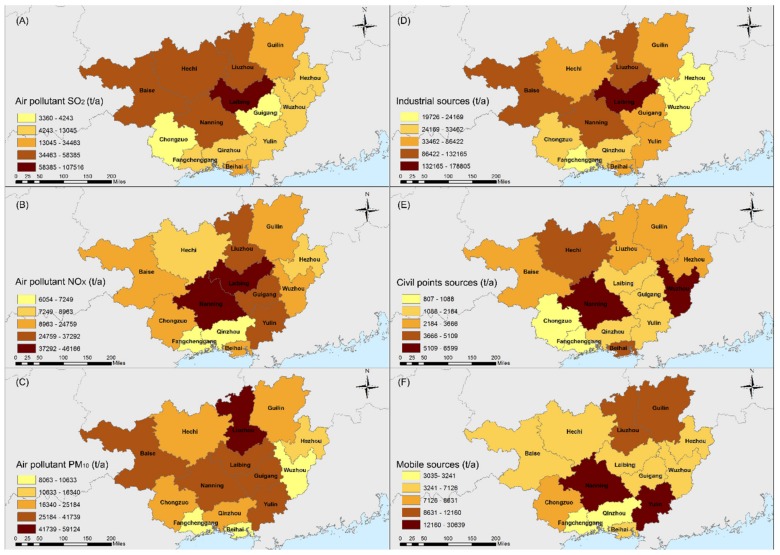
Major air pollutants (**A**–**C**) and different sources (**D**–**F**) in 14 cities of Guangxi Province.

Figure 3 shows the composition of the air pollutants (PM_10_, SO_2_, and NOx) in 14 cities in Guangxi. The total amount of air pollutant in Guangxi was approximately 1,163,096 t/a. Industrial sources contributed the highest percentage of the total air pollution (Figure 3D–F), including approximately 95% of the SO_2_, 91% of the PM_10_, and 61% of the NOx [35]. The cities with the most industrial pollutants were Laibing, Liuzhou, Nanning, and Baise. The civil points and mobile sources contributed less to air pollutants in Guangxi (Figure 3E–F). Nanning, Wuzhou, Hechi and Beihai had higher civil points pollution sources, whereas Nanning, Yulin, Liuzhou and Guilin accounted for higher mobile sources. It was noted that the civil sources of each city were relatively low and the differences between different cities were not significant. In Liuzhou and Laibing, the PM_10_, SO_2_ and NOx pollution mainly came from industrial pollution, demonstrating a serious industrial pollution problem. As for Nanning, although the industrial pollution contributed to the high PM_10_, SO_2_, and NOx levels, mobile sources were also an important contributor to the pollution problem. 

### 3.3. Spatial Distribution of Longevity Indicators in Guangxi

**Figure 4:**
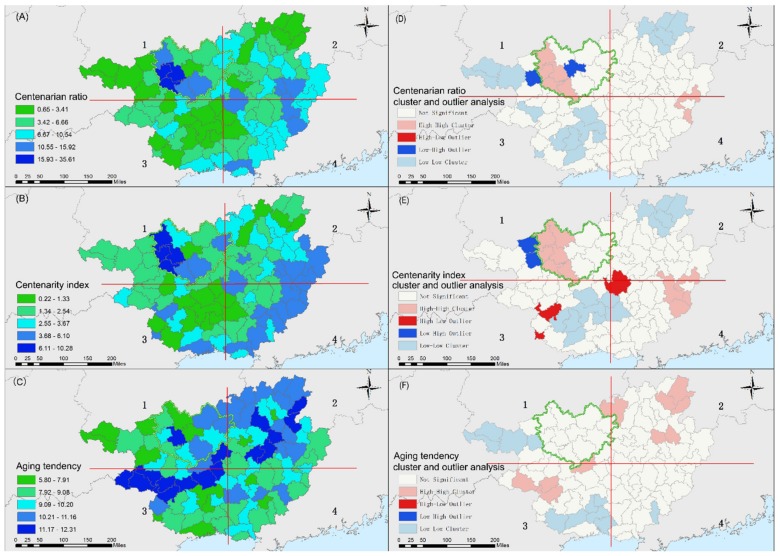
Spatial distribution of the three longevity indicators(**A**–**C**) and the results (**D**–**F**) of the cluster and outlier analysis.

Significant spatial differences were observed in the longevity indicators across the 109 counties and areas in Guangxi (Figure 4). Table 2 reveals significant differences among the centenarian ratios for counties in Guangxi, which had a standard deviation of 5.81 per 100,000 inhabitants. Fewer differences were found between the centenarity index and aging tendency, which had standard deviations of 1.79 per 100,000 inhabitants and 1.50 per 100,000 inhabitants, respectively. The areas with the highest centenarian ratios were situated in the central area of the 1st quadrant in the southwestern area of Hechi City (Figure 4A). These areas included the counties of Bama (36 per 100,000 inhabitants), Donglan (31 per 100,000 inhabitants), and Fengshan (28 per 100,000 inhabitants), which accounted for the significant longevity areas in Hechi City. Areas with a relatively low centenarian ratio were scattered in the 3rd quadrant. Similar to the distribution of the centenarian ratio, the northwestern areas had the highest centenarity index and represented significant longevity areas (Figure 4B), including Bama (10.28 per 100,000 inhabitants), Donglan (6.75 per 100,000 inhabitants), Fengshan (9.18 per 100,000 inhabitants), and Tiane (7.72 per 100,000 inhabitants), which were all located inside the 1st quadrant. In contrast, the higher values of the aging tendency were dispersed in the southwestern regions of the 3rd quadrant and the northeastern regions of the 2nd quadrant (Figure 4C), which included the urban areas of major cities, such as Nanning, Liuzhou, Laibing and Guilin.

According to the cluster and outlier analysis (Figure 4D,E), the centenarian ratio and centenarity index presented similar patterns in Guangxi, and they were highly clustered in the southwestern area inside Hechi City, especially in its longevity areas, including Tian’e, Bama, Donglan, Fengshan, and Dahua. In addition, both of these indicators were highly clustered in part of Wuzhou. In contrast, the centenarian ratio and centenarity index showed low clustering in major cities, such as Nanning and Guilin. On the other hand, the aging tendency showed high clustering in the southwestern and northeastern regions of Guangxi in areas mainly located in major cities (Figure 4F) such as Chongzuo, Guilin, Nanning, and some areas in Liuzhou. However, the aging tendency did not show high clustering in areas inside Hechi City. 

### 3.4. Relationship Between Air Pollutants and Longevity Indicators

In Table 3, the Geographically weighted regression(GWR) results showed that SO_2_, PM_10_, and NOx were negatively correlated with the longevity indicators individually. There are very similar R^2^ in the regression analysis between Centenarian ratio and SO_2_, PM_10_, and NOx and between Centenarian index and SO_2_, PM_10_, and NOx. The R^2^ is higher (up to 46%) in the regression analysis between aging tendency and SO_2_, PM_10_, and NOx. Based on the results in Section 3.1, Section 3.2 and Section 3.3, the longevity indicators in Hechi City showed anegative relationship with the air pollutants. The longevity areas in Hechi city recorded lower levels of air pollutants, especially in southwestern areas. The good air quality in these areas might be one of the important contributors contributed to the longevity in the city. In contrast, urban areas in cities such as Liuzhou, Nanning and Laibing, longevity levels were lower compared with Hechi city and three air pollutants indicators were higher, which indicated that air pollutants could be one of the negative contributors to longevity. Regarding air pollutant concentrations, the PM_10_ air pollutants were mainly distributed in the urban areas of Liuzhou, Laibing, Nanning, Baise, Guigang, and Yulin, subsequently. The SO_2_ air pollutants were mainly distributed in the urban areas of Liuzhou, Laibing, Nanning, southern areas in Baise and northeastern areas in Hechi. The NOx air pollutants were distributed in the urban areas of Liuzhou, Laibing, Nanning, Yulin, and Guigang. 

## 4. Discussion 

### 4.1. Spatial Distribution of Air Pollution in Guangxi and Its Origins

The areas with the most air pollutants were mostly in the central regions of the province, such as Liuzhou, Laibing, Nanning and Guigang, especially the industrial cities of Liuzhou and Laibing. These cities have relatively developed economies and dense populations, which contributed to air pollution. For example, Liuzhou and Laibing represent the largest industrialized centers and account for approximately 30% of the total industry in Guangxi [28]. Liuzhou showed the highest percentage of all three air pollutants. Liuzhou’s major industries include steel and machinery, chemical, automotive, and cement manufacturing [28]. The main industries of Laibing include thermal power generation, metal smelting, and cane sugar manufacturing. These two cities have represented the industrialization center of Guangxi since China’s Opening-up and Reform policy. The extensive industries in Liuzhou and Laibing consume a considerable amount of coal, and coal consumption is an important contributing source of SO_2_ and PM_10_. In addition, Nanning, the capital city of Guangxi, accounts for approximately 13% of the total industry in Guangxi and is the center of trade and a transportation hub between southwestern China and Southeast Asian countries. The developed economy, dense population, industrial and transportation infrastructure in this city have resulted in higher air pollution. 

The SO_2_ air pollution in Hechi was high. Industrial activities in Hechi include nonferrous metal mining and ore smelting, which are mainly concentrated in the northeastern part of Hechi, namely, the areas of Nandan and Huanjiang, and the urban areas of Hechi. In contrast, the areas with less air pollution were mainly situated in the in the southwestern part of Hechi, namely, the areas of Dahua, Fengshan, Bama, Donglan, and Du’an. These sites are located in isolated mountainous areas, where the economic development and industrialization are relatively low [40]; thus, the air pollutants in those areas were relatively low. 

### 4.2. Spatial Distribution of the Longevity Indicators in Guangxi

The population older than 90 years old (indicated by the centenarian ratio and centenarity index) in Guangxi was mainly located in the southwestern area of Hechi City. These areas were situated in comparatively remote zones with little economic or industrial development [29,40,41]. Many longevity studies have stated that elderly populations, especially those of the most elderly population, tended to be more affected by a better natural environment than by the economy and industrialization [40,41]. However, the northeastern part of Hechi City had lower centenarian ratios and centenarity indexes, which may have been due to the long-term environmental pollution from nonferrous metal exploitation. The local soil environment, river system, and underground water system in these parts of Hechi City might have contaminated by these activities to some extent.

The aging population older than 65 (aging tendency) tended to be distributed in the urban areas of the major cities in Guangxi, such as Nanning, Liuzhou, and Guilin. Many studies have maintained that good industrialization and economic development could support a high percentage of the elderly population, especially the younger aging population, in areas such as the Beijing-Tianjin, Yangtze River Delta and Pearl River Delta [2]. These areas with a well-developed economy could offer improved medical facilities, a comprehensive education system, and better social security systems; together, these benefits could increase the life expectancy and longevity in these regions [2,27]. Additionally, socioeconomic factors, such as education, infrastructural conditions, and health care, have helped maintain the relatively high percentage of individuals over 65 years old.

### 4.3. Associations Between Air Pollution and Regional Longevity

The mechanisms for regional longevity are comprehensive and include factors related to genetic makeup [4,5,6], the environment [7,8], and socioeconomic conditions [2,3,10]. Our research found that all air pollutants tested were negatively correlated with longevity indicators. Previous studies have revealed that SO_2_ and PM_10_ are significantly related to adverse effects on human health [42]. For example, a substantial association has been observed between an intense increase in the SO_2_ concentration and cardiovascular diseases [43]. PM_10_ has also been found to play an important role in cardiovascular and respiratory conditions [44]. Previous studies have also shown that certain groups of people, especially the elderly population were more likely to be impacted from disease resulting from increases in PM_10_ [26]. NOx exposure in the air could have negative impacts on human health [45,46], as it could contribute to the disease rate in the cardiovascular and respiratory systems of humans. 

More importantly, according to statistics, cluster and outlierand GWR analysis results, we found that the PM_10_, SO_2_, and NOx air pollution in the urban areas of Nanning, Liuzhou, Laibing, and Guigang, located in the central areas of Guangxi, was one of the negative contributors to the regional longevity in this province. PM_10_ air pollution in the urban areas of Liuzhou, Laibing, Nanning, Guigang, and Yulin mainly came from industrial sources, which contributed over 90% of the PM_10_ air pollution in these areas [35]. The SO_2_ air pollution in the urban areas of Liuzhou, Laibing, Nanning, Guilin, Baise and Hechi were also contributed by industrial sources and accounted for over 90% of the contribution. In addition, for the NOx air pollution in the urban areas of Liuzhou, Laibing, Nanning, Yulin and Guigang, industrial sources accounted for 60% and mobile sources accounted for approximately 30%. 

### 4.4. Implication for Aging Society Management

Based on the key findings from this study, to maintain a healthy aging society in Guangxi Province, the government will first need to reduce the PM_10_ and SO_2_ pollution and then NOx air pollution, because these areas contributed most negatively to the regional longevity at a provincial level. Because PM_10_ and SO_2_ air pollution were mainly contributed by industrial source emissions, the government will need to first focus on reducing the intensity of industrial sources by requiring extensive industries with higher air pollution upgrades and transform to intensive industries in the urban areas of the cities of Liuzhou, Laibing, Nanning, Guigang, and Yulin to reduce emissions and implement environmental policies to restrict emissions. In addition, for NOx control, reduce the mobile sources via the supervision of cargo and passenger vehicle use in the urban areas of Liuzhou, Laibing, Nanning, Yulin, and Guigang. The government should place more emphasis on the cities of Liuzhou, Laibing, and Nanning because they were all ranked among the top three cities in air pollutant emissions and accounted for considerable industrial sources pollution throughout the entire province. In addition, the “longevity city” Hechi had relatively higher SO_2_ air pollution in its northeastern areas. The local government should focus on restricting and improving the control of the local nonferrous metal industries in the affected areas to reduce industrial source emissions and maintain the high average longevity level in this city.

## 5. Conclusions

This study investigated the relationship between air pollutants and regional longevity at a provincial scale in Guangxi. We found that the spatial differences in the air pollution sources (SO_2_, PM_10_ and NOx) existed in the study area. The air pollutants PM_10_, SO_2_, and NOx had negative health impacts on the aging population. The PM_10_, SO_2_, and NOx air pollutants mainly came from industrial sources in the cities of Liuzhou, Laibing, Nanning, Baise, Hechi, Guigang, and Yulin. To maintain and improve the regional longevity, the government should focus on improving the intensive industries to reduce the industrial sources emission in the middle of Guangxi, particularly in Liuzhou, Laibing, Naning and Guigang. Implementation of policies to reduce industrial source emissions may require the revision of air pollution guidelines by relevant scientific agencies. In addition, the management and control of traffic pollution sources should be strengthened in Liuzhou, Laibing, Nanning, Yulin and Guigang.

## Figures and Tables

**Table 1:** Descriptive statistics of the air pollutants.

Indicators	Number	Minimum	Maximum	Mean	SD
SO_2_ t/a	109	50	22,410	2329	3683
NOx t/a	109	80	17,820	1839	3060
PM_10_ t/a	109	130	10,620	2061	2013

**Table 2:** Descriptive statistics of the longevity indicators.

Indicator	Number	Minimum	Maximum	Mean	SD
Centenarian ratio	109	0.65	35.61	6.82	5.81
Centenarity index	109	0.22	10.28	2.88	1.79
Aging tendency	109	5.7	12.31	9.65	1.5

**Table 3:** Summary of the GWR model of longevity indicators and air pollutants.

Dependent Variable	Independent Variable	β	R^2^	Adjust R^2^
Centenarian ratio	SO_2_	−0.163	51	37
	PM_10_	−0.256	52	39
	NO_X_	−0.176	49	35
Centenarity index	SO_2_	−0.129	46	31
	PM_10_	−0.199	44	30
	NOx	−0.127	43	29
Aging tendency	SO_2_	−0.035	57	41
	PM_10_	−0.046	50	34
	NOx	−0.055	60	46

## References

[1] United Nations, Department of Economic and Social Affairs, Population Division (2015). World Population Aging.

[2] Wang L., Li Y., Li H., Holdaway J., Hao Z., Wang W., Krafft T. (2016). Regional aging and longevity characteristics in China. Arch. Gerontol. Geriatr..

[3] Wang S., Luo K., Liu Y., Zhang S., Lin X., Ni R., Gao X. (2015). Economic level and human longevity: Spatial and temporal variations and correlation analysis of per capita GDP and longevity indicators in China. Arch. Gerontol. Geriatr..

[4] Christensen K., Vaupel J.W. (1996). Determinants of longevity: Genetic, environmental and medical factors. J. Intern. Med..

[5] Kenyon C.J. (2010). The genetics of ageing. Nature.

[6] Govindaraju D., Atzmon G., Barzilai N. (2015). Genetics, lifestyle and longevity: Lessons from centenarians. Appl. Transl. Genom..

[7] Magnolfi S.U., Noferi I., Petruzzi E., Pinzani P., Malentacchi F., Pazzagli M., Marchionni N. (2009). Centenarians in Tuscany: The role of the environmental factors. Arch. Gerontol. Geriatr..

[8] Lv J., Wang W., Li Y. (2011). Effects of environmental factors on the Longevous people in China. Arch. Gerontol. Geriatr..

[9] Magnolfi S., Petruzzi E., Pinzani P., Malentacchi F., Pazzagli M., Antonini F. (2007). Longevity index (LI%) and centenarity index (CI%): New indicators to evaluate the characteristics of aging process in the Italian population. Arch. Gerontol. Geriatr..

[10] Deng Q., Wei Y., Zhao Y., Han X., Yin J. (2018). Understanding the Natural and Socioeconomic Factors behind Regional Longevity in Guangxi, China: Is the Centenarian Ratio a Good Enough Indicator for Assessing the Longevity Phenomenon?. Int. J. Environ. Res. Public Health.

[11] Liu Y., Luo K., Lin X., Gao X., Ni R., Wang S., Tian X. (2014). Regional distribution of longevity population and chemical characteristics of natural water in Xinjiang, China. Sci. Total Environ..

[12] Wang W., Yang L., H Z. (2014). Integrated Analysis on Regional Development, Environment and Health in China.

[13] Zhang J., Mauzerall D.L., Zhu T., Liang S., Ezzati M., Remais J.V. (2010). Environmental health in China: progress towards clean air and safe water. Lancet.

[14] Cai J., Yu S., Pei Y., Peng C., Liao Y., Liu N., Ji J., Cheng J. (2018). Association between Airborne Fine Particulate Matter and Residents’ Cardiovascular Diseases, Ischemic Heart Disease and Cerebral Vascular Disease Mortality in Areas with Lighter Air Pollution in China. Int. J. Environ. Res. Public Health.

[15] Guan W., Zheng X., Chung K., Zhong N. (2016). Impact of air pollution on the burden of chronic respiratory diseases in China: Time for urgent action. Lancet.

[16] Guo Y., Li S., Tawatsupa B., Punnasiri K., Jaakkola J.J., Williams G. (2014). The association between air pollution and mortality in Thailand. Sci. Rep..

[17] Wen M., Gu D. (2012). Air pollution shortens life expectancy and health expectancy for older adults: The case of China. J. Gerontol. Ser. A Biol. Sci. Med. Sci..

[18] Liu C., Yin P., Chen R., Meng X., Wang L., Niu Y., You J. (2018). Ambient carbon monoxide and cardiovascular mortality: A nationwide time-series analysis in 272 cities in China. Lancet Planet. Health.

[19] Li T., Zhang Y., Wang J., Xu D., Yin Z., Chen H., Kinney P.L. (2018). All-cause mortality risk associated with long-term exposure to ambient PM2.5 in China: A cohort study. Lancet Public Health.

[20] Costa S., Ferreira J., Silveira C., Costa C., Lopes D., Relvas H., Borrego C., Roebeling P., Miranda A.I., Teixeira J.P. (2014). Integrating health on air quality assessment--review report on health risks of two major European outdoor air pollutants: PM and NO(2). J. Toxicol. Environ. Health B Crit. Rev..

[21] Shah A.S., Langrish J.P., Nair H., McAllister D.A., Hunter A.L., Donaldson K., Mills N.L. (2013). Global association of air pollution and heart failure: A systematic review and meta-analysis. Lancet.

[22] Wong C.M., Vichit-Vadakan N., Kan H., Qian Z. (2008). Public health and air pollution in Asia (PAPA): A multicity study of short-term effects of air pollution on mortality. Environ. Health Perspect..

[23] Raaschou-Nielsen O., Andersen Z.J., Beelen R., Samoli E., Stafoggia M., Weinmayr G., Xun W.W. (2013). Air pollution and lung cancer incidence in 17 European cohorts: Prospective analyses from the European Study of Cohorts for Air Pollution Effects (ESCAPE). Lancet Oncol..

[24] Bowe B., Xie Y., Li T., Yan Y., Xian H., Al-Aly Z. (2018). The 2016 global and national burden of diabetes mellitus attributable to PM 2.5 air pollution. Lancet Planet. Health..

[25] Apte J.S., Brauer M., Cohen A.J., Ezzati M., Pope C.A. (2018). Ambient PM2.5 reduces global and regional life expectancy. Environ. Sci. Technol. Lett..

[26] Wang L., Wei B., Li Y., Li H., Zhang F., Rosenberg M., Yang L., Huang J., Krafft T., Wang W. (2014). A study of air pollutants influencing life expectancy and longevity from spatial perspective in China. Sci. Total Environ..

[27] Song W., Li Y., Hao Z., Li H., Wang W. (2016). Public health in China: An environmental and socio-economic perspective. Atmos. Environ..

[28] Guangxi Statistical Bureau (2010). Guangxi Statistical Yearbook.

[29] Li Y., Li H., Wang W., Xiao Z. Longevity and its Environment in Hechi, Guangxi, China. Proceedings of the 5th International Conference on Population Aging and Longevity.

[30] Guangxi Zhuang Autonomous Region Bureau of Statistics, Office for Sixth Population Census of Guangxi Zhuang Autonomous Region (2012). Tabulation on the 2010 Population Census of Guangxi Zhuang Autonomous Regions.

[31] The Commission of China’s First Pollution Sources Census (2011). Data Set of the First National General Survey of Pollution Sources.

[32] The Commission of China’s First Pollution Sources Census (2011). Technology Report of National General Survey of Pollution Sources.

[33] The General Office of Guangxi Zhuang Autonomous Regions (2010). The first General Survey of Pollution Sources in Guangxi.

[34] Department of Environmental Protection of Guangxi Zhuang Autonomous Region (2011). Guangxi Environmental Year Book.

[35] Chinese Research Academy of Environmental Science, Guangxi Research Academy of Environmental Science of Guangxi Zhuang Autonomous Regions (2011). The Research Report of Atmosphere Environmental Capacity of Guangxi Zhuang Autonomous Region.

[36] Huang R. (2009). Assessing Accuracy in Age Reporting in China’s Population Census. Popul. Res..

[37] Cluster and Outlier Analysis (Anselin Local Moran’s I). https://pro.arcgis.com/en/pro-app/tool-reference/spatial-statistics/cluster-and-outlier-analysis-anselin-local-moran-s.htm.

[38] Mitchell A. (2005). The ESRI Guide to GIS Analysis.

[39] Brunsdon C., Fotheringham A.S., Charlton M.E. (2002). Geographically Weighted Regression: The Analysis of Spatially Varying Relationships.

[40] Deng Q., Chen L., Wei Y., Li Y., Han X., Liang W., Yin J. (2018). Understanding the Association between Environmental Factors and Longevity in Hechi, China: A Drinking Water and Soil Quality Perspective. Int. J. Environ. Res. Public Health.

[41] Qin J., Yu G., Xia T., Li Y., Liang X., Wei P., Long B., Lei M., Wei X., Tang X. (2017). Spatio-Temporal Variation of Longevity Clusters and the Influence of Social Development Level on Lifespan in a Chinese Longevous Area (1982–2010). Int. J. Environ. Res. Public Health.

[42] Liu Y., Chen X., Huang S., Tian L., Lu Y., Mei Y., Ren M., Li N., Liu L., Xiang H. (2015). Association between Air Pollutants and Cardiovascular Disease Mortality in Wuhan, China. Int. J. Environ. Res. Public Health.

[43] Amsalu E., Guo Y., Li H., Wang T., Liu Y., Wang A., Liu X., Tao L., Luo Y., Zhang F. (2019). Short-term effect of ambient sulfur dioxide (SO2) on cause-specific cardiovascular hospital admission in Beijing, China: A time series study. Atmos. Environ..

[44] Lee B.J., Kim B., Lee K. (2014). Air Pollution Exposure and Cardiovascular Disease. Toxicol Res.

[45] Boningari T., Smirniotis P.G. (2016). Impact of nitrogen oxides on the environment and human health: Mn-based materials for the NOx abatement. Curr. Opin. Chem. Eng..

[46] Latza U., Gerdes S., Baur X. (2009). Effects of nitrogen dioxide on human health: Systematic review of experimental and epidemiological studies conducted between 2002 and 2006. Int. J. Hyg. Environ. Health.

